# Tropical Diseases

**Published:** 1899-01

**Authors:** 


### Books and Magazines.

Tropical Diseases.—A Manual of the Diseases of Warm Climates. By Patrick Manson, M. D., L. L. D. (Aberd.); Fellow of the Royal College of Physicians, London; Physician to the Seaman's Hospital Society; Lecturer on Tropical Diseases at St. George's Hospital and Charing Cross Hospital Medical Schools; Medical Advisor to the Colonial Office and Crown Agents for the Colonies. With 88 illustrations and two colored plates. William Wood & Co. 1898. Pages, 603. Price, cloth, $3.50 net.

The perusal of the present work again calls attention to the fact often pointed out before that our own (American) text books on medicine are deficient in their discussion of diseases incident to the tropics. It is not, perhaps, good taste for a Southern physician to call attention to this deficiency, for the fault lies at our doors, but perhaps if attention was sufficiently called to the situation some talented Southern physician will yet put in enduring form the knowledge gained from experience in treating diseases peculiar to the South.

To prove that this deficiency is not imaginary, I will state that it was my fortune to practice medicine for several years in the Yazoo-Mississippi delta in Mississippi. I do not believe there is a more highly cultured medical profession in any agricultural community in the United States than is to be found there, yet I never heard a physician there appeal to a text book upon any question of treatment of purely Southern diseases. They are not recognized as authority, because they are, so far as diseases indigenous to the South are concerned, written from the laboratory and not from the clinical standpoint.

The work under consideration bears a very favorable contrast to these. It is written by a man who has had personal experience in the East Indies, and in addition to this, by his official position he has direct access to the vast stores of knowledge collected by the medical officers of the British army in almost every land under the sun.

The work is what might have been expected from such a source. In observation and deduction it corresponds almost identically to the opinions held by the best physicians of our own section.

Its discussion of dengue is especially satisfactory. Malaria, of course, comes in for the largest share of attention, and the text seems almost personally familiar, so closely does it correspond to our own knowledge of the subject. Tropical liver abscess, aestivo-autumnal fever, dysentery, yellow fever, cholera, filariasis and leprosy are among the leading subjects discussed. In addition to these all the rarer forms of tropical diseases not occurring in this country are amply discussed. If, however, our armies are to be sent to the uttermost ends of the earth, as seems probable, we had better begin to familiarize ourselves with these also.

The book is very valuable. I. J. J.

